# Dietary methionine source and level affect hepatic sulfur amino acid metabolism of broiler breeder hens

**DOI:** 10.1111/asj.12882

**Published:** 2017-08-04

**Authors:** Jianmei Wan, Xuemei Ding, Jianping Wang, Shiping Bai, Huanwei Peng, Yuheng Luo, Zhuowei Su, Yue Xuan, Keying Zhang

**Affiliations:** ^1^ Animal Nutrition Institute Sichuan Agricultural University Chengdu Sichuan China

**Keywords:** methionine, methionine hydroxyl analogue, plasma free amino acid, sulfur amino acids metabolism

## Abstract

A study was carried out to investigate the effects of dietary methionine source and level on plasma free amino acids patterns and the expression of genes involved in hepatic methionine metabolism in broiler breeders. A total of 2184 broiler breeders were assigned to 13 dietary treatments, with eight replicates per treatment. The 13 treatments included one control group and 12 additional treatments employing two sources and six levels (0.05, 0.10, 0.15, 0.20, 0.25 and 1.00%). Higher plasma methionine concentration was measured for DL‐methionine (DLM) treated hens. Plasma alanine concentration was linearly increased as DLM or 2‐hydroxy‐4‐(methylthio) butanoic acid (HMTBA) supplementation level increased. There was a linear increase in concentrations of tyrosine, valine, glycine and serine as dietary DLM supplementation level increased. Hens treated with DLM had higher relative expression of *ADA* than those fed HMTBA. The expression of *MS*,*ADA*,*SAHH* and *MAT2A* changed quadratically as HMTBA supplementation level increased, while the expression of *GNMT* and *SAHH* changed quadratically as DLM supplementation level increased. In conclusion, the effects of HMTBA on plasma free amino acid patterns and the expression of hepatic genes involved with methionine are different from DLM.

## Introduction

Amino acid supplementation has been practiced as one means of improving the utilization of dietary proteins. Methionine is often considered to be the first limiting amino acid for laying hens and supplementation of methionine is important to optimize the performance and health of layers. Considerable evidence in the literature supports the fact that 2‐hydroxy‐4‐(methylthio) butanoic acid (HMTBA), and 2‐hydroxy‐4‐(methylthio) butanoic acid‐calcium salts (HMTBA‐Ca) can replace DL‐methionine (DLM) as an effective methionine source in the diet of broilers (Sauer *et al*. 2008), pigs (Kim *et al*. 2006) and laying hens (Liu *et al*. 2005; Bunchasak *et al*. 2012).

For animals, four main pathways are responsible for methionine metabolism, including: (i) protein synthesis; (ii) transmethylation to form S‐adenosylmethionine (SAMe), a primary methyl donor that participates in the methylation process to form metabolites such as creatine, phosphatidylcholine, nucleic acids, proteins, and polyamine; (iii) deamination to form oxaloacetate, and then further transformed into glutamate, glutamine, proline, arginine, aspartate, asparagine, alanine, serine and glycine; (iv) transsulfuration to form L‐cysteine, which in turn is also a precursor for protein synthesis and can be incorporated into glutathione (GSH) or transformed into taurine (Mato *et al*. 2008; Bertolo & McBreairty 2013). Changes in the metabolism of methionine can influence the production of nutrients essential for proper functioning of the organs and systems such as liver, skeleton, muscle and cardiovascular (Miller & Kelly 1997). In mammals, up to 85% of all methylation reactions and as much as 48% of methionine metabolism occurs in the liver (Corrales *et al*. 2002), which suggests the importance of this organ in the regulation of blood methionine concentration.

The maintenance of the balance between the folate cycle, the methionine cycle, and the transsulfuration pathway in hepatic tissue relies on SAMe, GSH and the ratio of SAMe to S‐adenosylhomocysteine (SAH). If this balance was not reached, it could lead to liver injury via abnormal DNA synthesis, abnormal methylation reactions or oxidative stress. The SAMe:SAH ratio can be regulated by glycine N‐methyltransferase (GNMT) and SAH hydrolase (SAHH) which is involved in the hydrolysis of SAH to homocysteine (Hcy) and adenosine. Homocysteine can regenerate methionine by remethylation, or transform into cysteine and taurine in reactions requiring cystathionine β‐synthase (CBS). Adenosine can be metabolized by adenosine deaminase (ADA) or adenosine kinase (ADK). The removal of both adenosine and Hcy needs to be sufficiently rapid to maintain the flux in the direction of hydrolysis of SAH. The process of methionine regeneration by remethylation of Hcy in the liver can also be affected by dietary methionine levels via influencing the activities of betaine‐Hcy methyltransferase (BHMT), methyl‐THF reductase (MTHFR), methionine synthase (MS) and methionine synthase reductase (MSR) (Mato *et al*. 2008).

Due to the differences of chemical and physical characteristics between methionine sources, the bioavailability and physiological functions are not quite similar. The effects of methionine source and level on plasma free amino acids pattern and the hepatic methyl group metabolism of color‐feathered broiler breeder hens remain unclear. Therefore, the objective of the present study was to investigate the effects of dietary methionine source and level on the plasma free amino acids patterns and the expression of genes involved in hepatic methyl group metabolism in color‐feathered broiler breeder hens.

## Materials and methods

The animal experiment was conducted in accordance with guidelines approved by the Animal Health and Care Committee of Sichuan Agricultural University.

A 2 × 6 factorial completely randomized design with two methionine sources (DLM and HMTBA) and six dietary supplementation levels at 0.05, 0.10, 0.15, 0.20, 0.25 and 1.00% was used, and plus with a common basal group without methionine supplementation (Table 1). There were eight replicates per treatment and 21 hens per replicate of seven consecutive cages with three birds per cage (56 × 38 × 32 cm). A total of 2184 broiler breeder hens of Da Hen 699 (29 weeks old, a local breed in China) with similar initial average egg production performance were randomly divided into cages and treatments.

**Table 1:** Experimental design†

Treatment	Methionine source	Met supplementation level (%)	Addition of DLM products‡ (%)	Addition of HMTBA products§ (%)
1	Basal	0.000	0.000	—
2	DLM	0.050	0.051	—
3	DLM	0.100	0.101	—
4	DLM	0.150	0.152	—
5	DLM	0.200	0.202	—
6	DLM	0.250	0.253	—
7	DLM	1.000	1.010	—
8	HMTBA	0.050	—	0.057
9	HMTBA	0.100	—	0.114
10	HMTBA	0.150	—	0.172
11	HMTBA	0.200	—	0.229
12	HMTBA	0.250	—	0.286
13	HMTBA	1.000	—	1.144

†Calculated values. ‡Based on a content of 99% DL‐methionine (DLM) in the commercial product. §Based on a content of 88% DL‐2‐hydroxy‐4‐(methylthio) butanoic acid (HMTBA) in the commercial product.

The basal diet was formulated according to the breeder company's recommended nutrient levels except methionine level (Table 2). A large batch of the basal diet was processed and divided into portions for blending with the DLM or HMTBA at the highest methionine level on an equimolar basis at the expense of corn in the basal diet, then by mixing the basal and the highest methionine level diet to result in the other intermediate methionine level treatment diets. All the diets were in mash.

**Table 2:** Ingredient and nutrient concentration (%) of basal and highest Met level diets with DLM or HMTBA for breeder hens

Item	Basal	Met 1% (DLM)	Met 1% (HMTBA)
Corn, 7.8% CP	56.7	55.7	55.56
Wheat bran, 15.34% CP	9.1	9.1	9.1
Full‐fat soybean, 34.8% CP	17.6	17.6	17.6
Soybean meal, 43.0% CP	6.2	6.2	6.2
Limestone, large particle	6.00	6.00	6.00
Limestone, small particle	2.45	2.45	2.45
Dicalcium phosphate	0.92	0.92	0.92
NaCl	0.34	0.34	0.34
Choline hydrochloride, 70.0%	0.14	0.14	0.14
L‐lysine HCl	0.15	0.15	0.15
DLM, 99%	—	1.010	—
HMTBA, 88%	—	—	1.144
L‐threoinine	0.12	0.12	0.12
L‐tryptophan, 98.5%	0.045	0.045	0.045
Mineral premix†	0.20	0.20	0.20
Vitamin premix‡	0.035	0.035	0.035
Total	100	100	100
*Calculated values*			
CP	15.0	15.5	14.9
Calcium	3.50	3.50	3.50
Total P	0.52	0.52	0.52
Non‐phytate P	0.30	0.30	0.30
AMEn (MJ/kg)	11.3	11.3	11.3
Lysine	0.85	0.85	0.85
Methionine	0.23	1.23	1.23
Met + Cys	0.48	1.48	1.48
*Determined values*			
Crude protein	15.3	15.6	15.0
Lysine	0.91	0.97	0.94
Met	0.24	1.26	0.27
HMTBA^3^	0.009	0.009	1.003

†Mineral premix provided per kilogram of diet: copper (CuSO_4_·5H_2_O), 10 mg; iron (FeSO_4_·H_2_O), 60 mg; zinc (ZnSO_4_·H_2_O), 100 mg; manganese (MnSO_4_·H_2_O), 100 mg; iodine (KI), 1 mg; selenium (Na_2_SeO_3_), 0.3 mg. ‡Vitamin premix provided per kilogram of diet: vitamin A (retinyl acetate), 14 000 IU; vitamin D_3_, 4200 IU; vitamin E (DL‐*α*‐tocopheryl acetate), 63 IU; vitamin K, 5.25 mg; thiamin, 7 mg; riboflavin, 14 mg; pyridoxine, 7 mg; cyanocobalamin, 0.035 mg; niacin, 63 mg; pantothenicacid, 18.9 mg. Met, methionine; DLM, DL‐methionine; HMTBA, DL‐2‐hydroxy‐4‐(methylthio) butanoic acid; CP< crude protein; Cys, cysteine; AMEn, nitrogen corrected apparent metabolisable energy

All hens were reared in a house with an environmental temperature and relative humidity control system and free access to water with 16 h light program (04.00 to 20.00 hours). The daily feed was controlled with the feed allocation program as follows: 29–32 weeks: 130 g/hen/day; 33 weeks: 129 g/hen/day; 34–35 weeks: 128 g/hen/day; 36–37 weeks: 127 g/hen/day; 38–39 weeks: 126 g/hen/day and 40–43 weeks: 125 g/hen/day. The experiment lasted for 15 weeks. Egg production and hen mortality were recorded daily. Egg weights were measured weekly with all eggs produced during 2 consecutive days. Body weight was measured with three birds per replicate every 5 weeks.

Blood samples were collected from wing vein and the plasma samples were obtained with use of ethylenediaminetetraacetic acid as the anticoagulant and stored frozen (−20°C) until analysis. Plasma samples were precipitated with two volumes of 10% (w/v) 5‐sulfosalicylic acid, incubated at 4°C for 15 min, and centrifuged at 11 363 × *g*/min for 15 min at 4°C. The supernatant was collected in screw‐top cryovials and stored at 4°C. Plasma amino acids concentrations were determined with an automatic amino acid analyzer (L‐8800; Hitachi, Tokyo, Japan) with a ninhydrin reagent and lithium buffer system.

One hen per replicate was slaughtered by cervical dislocation and livers were sampled and snap‐frozen immediately in liquid nitrogen. Total RNA was extracted from liver tissues using RNAiso Plus reagent (Takara, Dalian, China). The complementary DNA (cDNA) was synthesized using the primeScript RT reagent kit (Takara, Dalian, China) and real‐time PCR was performed using the SYBR Premix Ex Taq (Takara, Dalian, China). All of these operations were carried out according to the manufacturer's instructions. Primer sequences are shown in Table 3. PCR reactions took place on an Applied Biosystems 7900HT Real Time PCR system (Applied Biosystems, Foster City, CA, USA) with the following programs: 95°C for 30 s, 40 cycles at 95°C for 5 s and 60°C for 34 s, 95 °C for 15 s, and a dissociation stage of 95°C for 15 s, 60°C for 1 min, 95°C for 15 s. Expression levels were normalized to beta‐actin and gene expression was calculated as 2^−ΔΔCt^ and expressed as relative fold change to basal group, as described by Livak and Schmittgen (2001). All real‐time PCRs were performed in triplicate in a 384‐well plate.

**Table 3:** Selected genes and primers used in this study

Gene‡	Accession number†	Orientation	Primer sequence (5΄→3΄)	Product size (bp)
β‐Actin	L08165	Forward Reverse	GAGAAATTGTGCGTGACATCA CCTGAACCTCTCATTGCCA	152
MAT1A	NM_001199519.1	Forward Reverse	TCATACCAGTGCGTGTCCAT CACACGATCCTTCAGGGTTT	95
MAT2A	XM_414497.4	Forward Reverse	CATTGCAGATGCATTCAACC TGACCTATCCCCAGCATCTC	129
BHMT	XM_414685.3	Forward Reverse	GGTGCTTCCATTGTTGGAGT CAGCTTGCAAACCCTCTTTC	88
PEMT	NM_001006164.1	Forward Reverse	CATGGCTGTAGGGACCTTGT CTCCATCAGGATGCCAAAGT	94
GNMT	XM_005512044.1	Forward Reverse	CATTGACCACCGCAACTATG TTACCAGCAGCACTGAGGTG	116
SAHH	XM_417331.4	Forward Reverse	ACTCCTTCACCAACCAGGTG GCTTCATCCAGCTTCTTTGG	100
MTHFR	XM_417645.4	Forward Reverse	GTGGGAGTGAGAGCTCCAAG AACTCCAGGGAGAACCACCT	130
MSR	XM_004935130.1	Forward Reverse	ATTGATGGTCTTTGGCTTGC TGCACTGACATTGTTGCTCA	81
MS	NM_001031104.1	Forward Reverse	TATGCTGCTGTCAGGTCTGG TGGCTACAGTCAGGGCTTCT	146
CBS	XM_416752.3	Forward Reverse	CTGGGATCTTGAAACCTGGA TCAGGACATCCACCTTCTCC	147
ADA	NM_001006290.2	Forward Reverse	GAAAGGAATTTCCGCATCAA GGCCACCACAGAGTTGTTTT	117
ADK	NM_001006501.1	Forward Reverse	TTCCCTGTGTTGGTGAGTGA TAATGACGCTGGCTGCATAG	148

†GenBank accession number. ADA, adenosine deaminase; ADK, adenosine kinase; BHMT, betaine‐homocysteine methyltransferase; GNMT, glycine N‐methyltransferase; ‡MAT1A, methionine adenosyltransferase 1A; MAT2A, methionine adenosyltransferase 2A; MS, methionine synthase; MSR, methionine synthase reductase; MTHFR, methyl‐THF reductase; PEMT, phosphatidylethanolamine N‐methyltransferase; SAHH, S‐adenosylhomocysteine hydrolase.

The data were subjected to a two‐way analysis of variance (ANOVA) (two methionine sources; seven supplementation levels of methionine) using the GLM procedures of SAS (SAS Institute Inc., Cary, NC, USA). The model included source, supplementation level, and source × level as main effects. Contrast statements were utilized to test the significance of both linear and quadratic terms for methionine supplementation level as explanatory factors of the response obtained. Least square means for treatments showing significant differences in the ANOVA were separated by Tukey's test. Statements of statistical significance of difference were based on the probability of a type I error set at *P *<* *0.05, and trends were accepted if 0.05 < *P *<* *0.10.

## Results

The mortality during the whole period was very low (0.60% as a total), and neither methionine source nor level affected the mortality rate (data not shown). Egg production, egg weight, feed conversion, settable eggs per hen produced and average daily gain (ADG) were not affected by the methionine source or level or the interaction of methionine source and level (Table 4) (*P *>* *0.05).

**Table 4:** Effects of methionine source and level on production performance of broiler breeder hens (29−43 weeks of age)

Treatment	Egg production (%)	Egg weight (g/egg)	FCR (g/g)	ADG (g/hen/day)	Settable eggs (eggs/hen‐housed)
Basal	55.67	53.66	4.30	3.54	49.7
DLM‐0.05	52.30	53.47	4.25	3.97	44.5
DLM‐0.10	55.50	54.25	4.29	1.49	49.6
DLM‐0.15	55.87	53.49	4.48	1.28	48.9
DLM‐0.20	53.45	53.55	4.56	4.10	46.8
DLM‐0.25	52.52	53.99	4.32	2.78	47.5
DLM‐1.00	55.53	53.61	4.45	0.38	49.2
HMTBA‐0.05	53.65	53.99	4.51	3.92	47.5
HMTBA‐0.10	52.81	54.03	4.84	2.43	46.9
HMTBA‐0.15	49.93	53.90	4.22	3.34	44.2
HMTBA‐0.20	56.50	53.64	4.48	2.77	50.0
HMTBA‐0.25	53.46	53.98	4.39	2.37	47.3
HMTBA‐1.00	54.07	53.97	4.76	4.04	48.6
SEM	2.05	0.28	0.19	1.08	2.03
*P*‐value	0.548	0.679	0.457	0.333	0.510
Main effects		*P*‐value			
Source	0.483	0.253	0.504	0.676	0.756
Level	0.783	0.423	0.681	0.157	0.609
Source × level	0.235	0.777	0.198	0.437	0.256
*Contrast*					
DLM linear	0.422	0.628	0.575	0.630	0.760
DLM quadratic	0.529	0.921	0.258	0.150	0.974
HMTBA linear	0.763	0.878	0.730	0.417	0.682
HMTBA quadratic	0.215	0.681	0.232	0.983	0.227

FCR, feed conversion ratio; ADG, average daily gain; DLM, DL‐methionine; HMTBA, DL‐2‐hydroxy‐4‐(methylthio) butanoic acid.

The results of the effects of methionine source and level on plasma free amino acids concentration were shown in Table 5. Plasma methionine concentration was significantly affected by methionine source and level (*P *<* *0.01) and a significant interaction between source and level was observed (*P *<* *0.01). The plasma methionine concentration increased linearly as dietary DLM or HMTBA supplementation level increased, but methionine concentration rose more rapidly when DLM was supplemented (source × level, *P *<* *0.01). There were linear (DLM, *P *=* *0.03; HMTBA, *P *=* *0.02) increases for plasma alanine concentrations. There was also a linear increase for concentrations of valine (*P *=* *0.03), tyrosine (*P *=* *0.02), glycine (*P *=* *0.05), serine (*P *<* *0.01), aspartate (*P *=* *0.08) and leucine (*P *=* *0.06) as dietary DLM supplementation level increased. Plasma concentrations of aspartate (*P *<* *0.01) and glutamate (*P *=* *0.06) of hens treated with HMTBA was higher than those treated with DLM. There was no significant effect of methionine source and level on plasma concentrations of cysteine, taurine and cystathionine (*P *>* *0.10). When compared with the basal group, supplementing methionine at 1% in the diet significantly elevated plasma methionine concentration (3.82‐fold for DLM, 1.87‐fold for HMTBA) (*P *<* *0.05) and supplementing methionine using DLM as a source at 1% significantly increased plasma alanine concentration (*P *<* *0.05).

**Table 5:** Effects of methionine source and level on plasma free amino acids concentration of broiler breeder hens (mg/L)

Treatment	Met	Cys	Tau	Cysthi	Leu	Ile	Val	Ala	Phe	Pro	Gly	Thr	Ser	Tyr	Lys	Arg	His	Asp	Glu
Basal	12.03^d^	10.98	21.13	0.89	34.84	16.53	35.48	37.39^dc^	17.08	46.34	33.04	36.03	52.00	21.21	53.52	134.54	18.75	6.18	34.80
DLM‐0.05	12.42^d^	11.91	14.99	2.31	26.75	13.25	27.80	32.68^d^	14.41	41.73	29.20	41.64	52.59	17.73	44.09	101.21	16.36	4.58	27.18
DLM‐0.10	13.91^d^	15.51	27.24	4.18	30.72	15.42	32.23	37.71^dc^	16.62	46.73	33.89	43.39	57.94	23.06	59.82	126.76	18.33	4.83	37.93
DLM‐0.15	18.72^cd^	15.69	20.98	2.81	33.50	17.08	36.54	45.29abc	18.65	53.91	36.67	34.12	67.82	23.23	46.62	176.59	17.34	4.22	34.90
DLM‐0.20	20.14^cd^	13.61	19.24	1.44	34.43	16.52	36.17	43.66abc	16.98	58.76	35.57	55.03	67.90	22.38	47.61	157.16	17.79	6.75	33.58
DLM‐0.25	27.33^bc^	11.15	19.87	1.66	33.46	17.17	35.88	43.91abc	18.66	54.88	39.02	48.64	68.80	26.70	53.66	148.42	17.14	5.88	34.71
DLM‐1.00	76.96^a^	13.28	27.47	3.83	37.65	17.77	36.53	51.41^a^	19.53	58.92	36.63	41.12	66.62	29.18	48.55	160.48	21.70	5.73	37.95
HMTBA‐0.05	13.65^d^	11.31	23.07	1.97	33.12	16.40	34.25	37.86^dc^	18.53	46.84	33.82	35.35	64.26	22.72	49.69	123.85	17.11	6.02	36.09
HMTBA‐0.10	12.55^d^	15.00	19.44	1.86	33.09	16.30	35.21	40.06bcd	16.72	49.46	34.50	36.24	62.21	19.69	53.86	118.16	16.16	8.20	40.83
HMTBA‐0.15	15.04^d^	8.38	22.34	1.92	29.56	14.11	29.99	36.43^cd^	16.78	41.13	34.38	37.89	66.04	20.50	47.01	128.71	20.40	7.13	40.03
HMTBA‐0.20	15.48^d^	15.47	20.17	2.66	33.62	14.90	35.86	42.20abcd	19.00	51.93	37.16	40.36	55.10	22.36	57.65	147.45	20.25	7.22	35.11
HMTBA‐0.25	21.09^cd^	10.52	19.10	1.26	34.82	17.25	36.29	46.06abc	18.92	58.28	37.12	45.34	70.16	22.92	50.96	121.84	18.37	8.07	40.69
HMTBA‐1.00	34.74^b^	12.31	23.20	2.28	29.37	14.40	30.62	43.06abcd	18.16	41.73	34.32	38.32	66.06	24.47	58.07	151.64	19.29	7.56	39.17
SEM	3.37	2.39	2.94	1.46	2.91	1.72	3.42	3.46	1.26	8.00	2.90	5.64	6.32	2.37	6.36	36.73	2.18	1.17	4.44
*P*‐value	<0.01	0.45	0.15	0.47	0.35	0.73	0.61	0.02	0.20	0.65	0.66	0.25	0.23	0.11	0.81	0.98	0.83	0.09	0.51
*Source*																			
DLM	28.24^a^	13.53	21.63	2.71	32.75	16.20	34.19	42.44	17.48	52.49	35.16	43.99	63.61	23.71	50.06	145.10	18.11	5.33^b^	34.37
HMTBA	18.76^b^	12.16	21.22	1.99	32.26	15.56	33.70	40.95	18.02	48.23	35.22	38.92	63.97	22.11	52.87	131.94	18.60	7.37^a^	38.65
SEM	1.39	0.94	1.07	0.45	1.11	0.68	1.33	1.37	0.50	3.06	1.16	2.17	2.31	0.94	2.55	14.77	0.85	0.42	1.58
*Level*																			
	12.03^c^	10.98	21.13	0.89	34.84	16.53	35.48	37.39^cd^	17.08	46.34	33.04	36.03	52.00^b^	21.21^b^	53.52	134.54	18.75	6.18	34.80
0.05	13.04^c^	11.61	19.03	2.14	29.94	14.83	31.03	35.27^d^	16.47	44.28	31.51	38.49	58.43^ab^	20.23^b^	46.89	112.53	16.74	5.30	31.64
0.10	13.23^c^	15.26	23.34	3.02	31.90	15.86	33.72	38.88bcd	16.67	48.10	34.19	39.82	60.08^ab^	21.37^b^	56.84	122.46	17.24	6.51	39.38
0.15	16.88^c^	12.03	21.66	2.36	31.53	15.59	33.26	40.86abcd	17.71	47.52	35.53	36.00	66.93^a^	21.87^b^	46.81	152.65	18.87	5.67	37.46
0.20	17.81^c^	14.54	19.71	2.05	34.02	15.71	36.01	42.93abc	17.99	55.35	36.36	47.70	61.50^ab^	22.37^ab^	52.63	152.30	19.02	6.99	34.35
0.25	24.21^b^	10.84	19.48	1.46	34.14	17.21	36.09	44.98^ab^	18.79	56.58	38.07	46.99	69.48^a^	24.81^ab^	52.31	135.13	17.75	6.98	37.70
1.00	55.85^a^	12.79	25.34	3.06	33.51	16.08	33.57	47.24^a^	18.85	50.32	35.47	39.72	66.34^a^	26.82^a^	53.31	156.06	20.49	6.64	38.56
SEM	2.48	1.63	1.91	0.86	1.99	1.23	2.39	2.45	0.89	5.48	2.07	3.84	4.17	1.70	4.56	26.72	1.51	0.75	2.87
Main effects												*P‐*value							
Source	<0.01	0.30	0.78	0.20	0.75	0.50	0.79	0.43	0.43	0.32	0.97	0.09	0.91	0.22	0.43	0.52	0.68	<0.01	0.06
Level	<0.01	0.34	0.12	0.51	0.56	0.80	0.61	<0.01	0.22	0.51	0.27	0.13	0.04	0.07	0.54	0.77	0.49	0.41	0.33
Source×level	<0.01	0.45	0.06	0.65	0.12	0.35	0.30	0.17	0.13	0.55	0.77	0.63	0.39	0.29	0.73	0.95	0.68	0.76	0.91
*Contrast*																			
DLM linear	<0.01	0.62	0.82	0.41	0.06	0.10	0.03	0.03	0.12	0.06	0.05	0.07	<0.01	0.02	0.76	0.25	0.87	0.08	0.34
DLM quadratic	0.08	0.06	0.40	0.55	0.27	0.41	0.30	0.21	0.35	0.39	0.60	0.57	0.40	0.73	0.86	0.29	0.60	0.77	0.19
HMTBA linear	<0.01	0.86	0.43	0.80	0.62	0.95	0.60	0.02	0.36	0.33	0.19	0.08	0.77	0.67	0.72	0.81	0.32	0.39	0.80
HMTBA quadratic	0.13	0.64	0.99	0.53	0.29	0.21	0.35	0.17	0.16	0.39	0.86	0.57	0.30	0.34	0.84	0.80	0.43	0.72	0.88

^a–d^Means within a column with no common superscript differ significantly (*P *<* *0.05). Cysthi, cystathionine, Tau, taurine; DLM, DL‐methionine; HMTBA, DL‐2‐hydroxy‐4‐(methylthio) butanoic acid.

**Figure 1:**
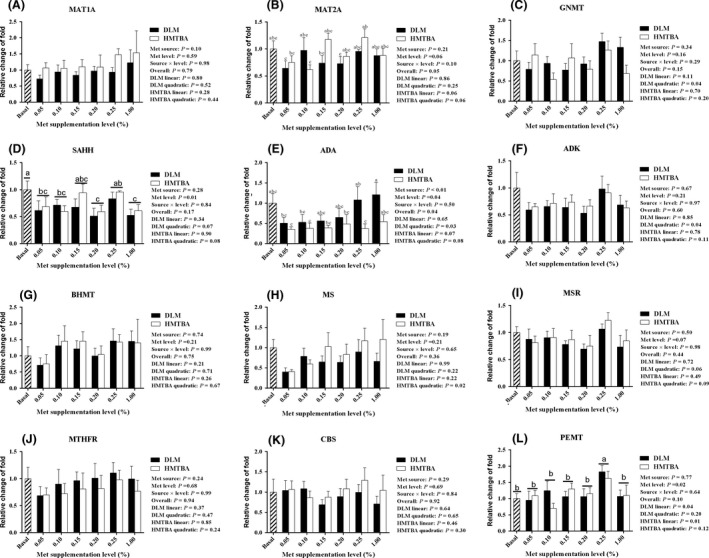
Effects of methionine source and level on the expression of genes involved in hepatic methyl group metabolism in broiler breeder hens. The expression of *MAT1A* (A)*, MAT2A* (B)*, GNMT* (C)*, SAHH* (D)*, ADA* (E)*, ADK* (F)*, BHMT* (G)*, MS* (H)*, MSR* (I)*, MTHFR* (J)*, CBS* (K)*,* and *PEMT* (L) genes was determined by quantitative RT‐PCR. The results are presented as an average relative fold change in the expression of each gene in the livers of hens fed the DL‐methionine (DLM) or 2‐hydroxy‐4‐(methylthio) butanoic acid (HMTBA) supplemental diet relative to that without any DLM or HMTBA, which was assigned a value 1. a–c, Different letters indicate statistical difference at *P *<* *0.05. ADA, adenosine deaminase; BHMT, betaine‐homocysteine methyltransferase; MAT1A, methionine adenosyltransferase 1A; MAT2A, methionine adenosyltransferase 2A; MS, methionine synthase; MTHFR, methyl‐THF reductase; SAHH, S‐adenosylhomocysteine hydrolase; CBS, cystathionine β‐synthase.

Figure 1 presents the results of relative expression of genes involved in hepatic sulfur amino acid metabolism. The relative expressions of *MAT2A* (*P *=* *0.06), *ADA* (*P *=* *0.07) and *PEMT* (*P *=* *0.01) were affected by HMTBA supplementation level. As HMTBA supplementation level increased, the expression of *MSR* (*P *=* *0.09), *MS* (*P *=* *0.02), *ADA* (*P *=* *0.08), *SAHH* (*P *=* *0.08) and *MAT2A* (*P *=* *0.06) changed quadratically (went down first and then went up). Hens treated with DLM had a higher (*P *<* *0.01) relative messenger RNA (mRNA) expression of *ADA* than hens treated with HMTBA. As DLM supplementation level increased, relative expression of *PEMT* increased linearly and the expression of *GNMT* (*P *=* *0.04), *MSR* (*P *=* *0.06) and *SAHH* (*P *=* *0.07) changed quadratically (went down first and then went up).

## Discussion

Methionine is usually the first limiting amino acid for laying hens and supplementation of DLM or HMTBA as methionine source is important to optimize the performance and health of layers. Effects of dietary methionine supplementation level on performance of broiler breeders has been documented by several studies (Bornstein *et al*. 1979; Bowmaker & Gous 1991; Harms & Russell 1998; Gomes *et al*. 2011). A range of methionine requirements from 321 (Bowmaker & Gous 1991) to 570 mg/hen/day (Bornstein *et al*. 1979) for broiler breeders has been reported. No response was obtained from increasing methionine intake from 399 to 436 mg/hen/day or 413 to 443 mg/hen/day (Harms & Russell 1998). The present study also showed that no response was obtained as methionine intake increased from 299 to 1599 mg/hen/day. Based on the values of methionine content of whole egg (3.646 mg/g egg) (Lunven *et al*. 1973) and the efficiency of dietary methionine for egg production (55.2%) (Nonis & Gous 2006), the methionine requirement for producing eggs is up to 661 mg/100 g egg, so the egg mass has critical effect on the methionine requirement of broiler breeders. The methionine required for maintenance was also estimated as suggested by Nonis and Gous (2006) and Fisher (1998) to be 115.6 mg/hen/day. In the present study, the egg mass ranged from 27–30 g/hen/day, so the total methionine requirement of broiler breeders was estimated to be 291.1–313.9 mg/hen/day, which was just slightly higher than the amount supplied by the basal diet (262.5–273 mg/hen/day). This may be the main reason there was no effects of methionine source and level on egg production performance of broiler breeder hens and no deficient symptom for hens fed basal diets. Adding methionine at 1% (from HMTBA or DLM) did not suppress the production performance of broiler breeders in the present study, which was not in line with those reports by Xie *et al*. (2007) and Wideman *et al*. (1994). Adding DLM at 1% or HMTBA at 1.13% by Xie *et al*. (2007) reduced average daily feed intake (ADFI) of ducks to 207.6 g/bird/day or 224.1 g/bird/day, respectively, but the ADFI was higher than that of broiler breeders in the present study (130 g/bird/day at peak). ADFI of commercial laying hens was significantly reduced by adding DLM at 1% or HMTBA at 1.13% when compared with the control group (Wideman *et al*. 1994). These results showed that broiler breeders can tolerate a higher methionine level than commercial laying hens.

Four main roles are responsible for methionine from diet, including: (i) protein synthesis; (ii) transmethylation to form SAMe, a primary methyl donor that participates in the methylation process to form metabolites such as creatine, phosphatidylcholine, nucleic acids, proteins and polyamine; (iii) deamination to form oxaloacetate, and then further transforms into nonessential amino acids (NEAA) such as glutamate, glutamine, proline, arginine, aspartate, asparagine, alanine, serine and glycine; (iv) methionine that has not been used in the liver is transported via blood to other tissues and utilized. The results of the present study showed that there was a significant effect of methionine source and level on hepatic methionine metabolism, and a significant difference was also observed for the effect on hepatic methionine metabolism between DLM and HMTBA. First, methionine source and level affect the profile of plasma free amino acids. Liver is the main site of methionine metabolism, and is also the main site for the synthesis of structural proteins and secreted proteins such as serum proteins and egg proteins. It provides amino acids to the peripheral tissues and plays an important role in regulating the plasma methionine concentration (Finkelstein 1990). Ohta and Ishibashi (1995) reported that plasma methionine concentration increased rapidly with increasing dietary DLM levels, and reduced by adding glycine (0.60%), but plasma glycine, threonine and serine concentrations were not affected in broilers fed with methionine excess (1.56%) diet. Glycine can alleviate methionine toxicity because glycine and threonine can be metabolized to serine which is required as a carbon skeleton source for the synthesis of cystathionine and regeneration of methionine from homocysteine (Girard‐Globa *et al*. 1972; Katz & Baker 1975). In the present study, plasma glycine, threonine and serine concentrations were increased as dietary DLM levels increased until large excess methionine (1%) was supplemented, in which the concentrations were reduced numerically. Studies (Lobley *et al*. 2001; Wang *et al*. 2001) have shown that the broiler's liver apparently metabolized 86% of the HMTBA that entered the liver. Most of the methionine produced is retained in the tissues for anabolic use. Only a small amount of synthesized methionine is released from the tissues of the liver. This synthesis and use within tissues accounts for the relatively small changes and lower plasma methionine concentration for breeders treated with HMTBA than their counterparts treated with DLM, although there was a linear change as dietary HMTBA level increased. Wu (2013) pointed out that excess methionine can be converted into oxaloacetic acid in the body, and then is involved in glycolipid metabolism or synthesis nonessential amino acids such as aspartate, asparagine, glutamate, glutamine, glycine and serine via deamination and transamination. In the present study, plasma alanine concentration increased linearly as dietary DLM or HMTBA level increased, but plasma concentrations of valine, glycine, serine and tyrosine increased linearly only as dietary DLM level increased. The results suggested that there was some difference in hepatic metabolism processes between DLM and HMTBA and the processes are affected by methionine source and level.

Although there are many factors that affect the profile of plasma free amino acids, and the biochemical mechanism involved in regulating the profile is complicated, the influence of methionine source and level on profile of plasma free amino acids of broiler breeders is at least related to the following two aspects. On the one hand, synthesis of methionine via HMTBA needs an amino donor. Rangel‐Lugo and Austic (1998) and Martín‐Venegas *et al*. (2006) found that a wide variety of amino acids can serve as substrates for conversion of HMTBA to methionine in chicken, but leucine, valine, alanine and glutamine were the preferred amino donors. On the other hand, methionine source and level affect hepatic methionine metabolism, which in turn affects the plasma concentration of those amino acids that are closely related to this process. Methionine adenosyltransferase 2A and methionine synthase are encoded by *MAT2A* and *MS*, respectively. Methionine adenosyltransferase is the limited enzyme that catalyzes methionine to form SAMe. SAMe is the methyl donor in many methylation reactions. Methionine synthase is an enzyme that links the methionine cycle and the folate cycle and catalyzes the remethylation reaction of homocysteine to generate methionine, which is one of the means of homocysteine elimination and methionine regeneration (Mato *et al*. 2008). In the cases of HMTBA as a source of methionine, the body may release signals of methionine deficiency, which may increase the expression of MS to enhance methionine regeneration and meanwhile reduce methionine consumption of methylation reaction. Studies using isotope tracers in broilers have shown that methionine synthase pathway is the main pathway for homocysteine remethylation to regenerate methionine, whereas betanine‐homocysteine methyltransferase pathway is a secondary pathway (Pillai *et al*. 2006a,b). Methylation of homocysteine by methionine synthase pathway requires methyltetrahydrofolate to provide a methyl group which is derived from the methylneogenesis pathway which requires the participation of serine and folate. In animals, threonine can be converted to glycine, and glycine, alanine and serine can be transformed into each other (Mato *et al*. 2008; Wu 2013). The increase of homocysteine remethylation will accelerate the consumption of homocysteine and SAMe. SAMe regulates the balance between the methionine cycle and folate cycle. SAMe is an allosteric activator of methionine adenosyltransferase III, the predominant liver isoenzyme that synthesizes SAMe, which is activated by methionine (del Pino *et al*. 2000). When the availability of methionine is low, hepatic SAMe content drops, releasing the inhibition of methyltetrahydrofolate and exerting on the synthesis of methionine through the methylneogenesis pathway to restore the normal levels of methionine. This may be the reason why the expressions of *MS* and *MAT2A* and the plasma concentrations of alanine and threonine increased linearly with the addition of HMTBA. In contrast, when the concentration of methionine is elevated using DLM as a source of methionine, hepatic SAMe content increases, activating methionine catabolism via the transmethylation and transsulfuration pathways and inhibiting methionine regeneration via the methylneogenesis pathway to restore the normal methionine content (Mato *et al*. 2008). The glycine methyltransferase encoded by the *GNMT* gene catalyzes the conversion of SAMe to SAH. This process requires the participation of glycine, and SAMe will donate its methyl group to a variety of methyl group receptors. SAHH catalyzes the hydrolysis of SAH to form adenosine and Hcy. Since this step is a reversible reaction, adenosine or Hcy needs to be rapidly removed in order for the reaction to proceed in the direction of Hcy formation. Adenosine deaminase encoded by the *ADA* gene catalyzes the hydrolysis of adenosine into inosine, thereby promoting the conversion of SAH to form Hcy (Mato *et al*. 2008). This indicated that with the increase of dietary DLM level, more methionine should be converted to Hcy to form other substances such as cysteine and taurine.

In conclusion, the data of the present study showed that the effects of HMTBA on plasma free amino acid patterns and the expression of hepatic genes involved in methionine metabolism are different from DLM.
